# Pulmonary Risk Stratification in Open Thoracoabdominal Aortic Aneurysm Repair

**DOI:** 10.3390/jcm15072623

**Published:** 2026-03-30

**Authors:** Jelle Frankort, Mohammed Al-Falahi, Andras Keszei, Bernhard Hruschka, Quentin Cappel, Christian Uhl, Alexander Gombert

**Affiliations:** 1 Department of Vascular Surgery, Medical Faculty, RWTH Aachen University, 52074 Aachen, Germany; 2 Department of Vascular Surgery, MUMC+, 6229 HX Maastricht, The Netherlands; 3 Centre for Translational & Clinical Research Aachen (CTC-A), University Hospital RWTH, 52074 Aachen, Germany

**Keywords:** thoracoabdominal aortic aneurysms, open surgical repair, pulmonary function testing

## Abstract

**Background/Objectives:** The aim of this study was to assess whether preoperative pulmonary function testing (PFT) is related to postoperative complications after open thoracoabdominal aortic aneurysm (TAAA) repair. **Methods:** This study was conducted as a retrospective cohort analysis of 205 patients undergoing open TAAA repair (2006–2024) with preoperative spirometry and body plethysmography with at least one value available. Patients were classified by ventilation patterns: obstructive (n = 85, 45.2%), restrictive (n = 26, 14.1%), and hyperinflation (n = 56, 30.3%). Primary endpoints included in-hospital mortality, pulmonary complications (pneumonia, ARDS), and multi-organ outcomes. Associations were analyzed using chi-square and Spearman correlation tests and multivariable linear regression adjusted for age, smoking status, COPD, emergency operation, and time period. **Results:** Postoperative pulmonary complications occurred in 126 patients (61.5%), including pneumonia (46.8%) and ARDS (15.1%). Reduced vital capacity and FEV_1_ expressed as a percentage of the lower limit of normal (%LLN) were related to postoperative pneumonia (*p* = 0.031 and *p* = 0.003) and ARDS (*p* = 0.038). Both obstructive and restrictive ventilation patterns were related to acute kidney injury after surgery (all KDIGO stage) (*p* = 0.044 and *p* = 0.043, respectively). Hyperinflation was related to atrial fibrillation (*p* = 0.039) and stroke (*p* = 0.034). FEV_1_ < 2.0 L was related to increased mortality risk (*p* = 0.037), and FEV_1_ < 1.5 L predicted acute kidney injury (*p* = 0.017), spinal cord ischemia (*p* = 0.035), and mortality (*p* = 0.023). Prolonged mechanical ventilation correlated with reduced preoperative lung function (VC %LLN ρ = −0.288, *p* = 0.002; FEV_1_ %LLN ρ = −0.286, *p* = 0.001). During median follow-up of 6.35 years, patients in the highest FEV_1_ quartile demonstrated substantially reduced long-term mortality (HR 0.27, 95% CI 0.10–0.73, *p* = 0.01). These associations between lower FEV_1_ and VC (expressed as %LLN) with pneumonia, ARDS, in-hospital mortality, and prolonged ventilation remained significant after multivariable analysis. **Conclusions:** Preoperative pulmonary function assessment may help identify TAAA patients at increased risk of postoperative complications and mortality. Combining percentage-predicted spirometry, ventilation patterns, and hyperinflation markers may support individualized treatment selection, prehabilitation, and perioperative monitoring based on each patient’s specific risk profile.

## 1. Introduction

Open thoracoabdominal aortic aneurysm (TAAA) repair is a complex surgical procedure associated with significant morbidity and mortality [1,2]. Postoperative pulmonary complications are a major concern, with reported rates ranging from 20–50% [3]. Identifying patients at high risk of respiratory complications is crucial for optimizing perioperative management and improving outcomes.

Preoperative pulmonary function testing (PFT) has been widely used to assess respiratory status and predict postoperative complications in various surgical populations [4,5,6]. Despite its general acceptance, there is limited information regarding its usefulness in the context of TAAA repair. Some research studies have reported correlations between suboptimal preoperative lung function and increased risk of postoperative pulmonary complications, while others have cast doubt on the predictive value of PFTs in this population [5]. Patients with pre-existing lung disease may be particularly susceptible to postoperative complications. Chronic obstructive pulmonary disease (COPD) has been identified as an independent risk factor for mortality and morbidity following TAAA repair [4]. Recent advances in operative techniques, especially endovascular approaches, have reduced the overall incidence of pulmonary complications [7]. However, open repair remains necessary for complex aneurysms in a small subgroup of patients where endovascular repair is not feasible, and respiratory failure continues to be a significant cause of prolonged intensive care unit stay and mortality in this population. Given the high-risk nature of TAAA repair and the potential for severe respiratory complications, there is a need to better define the role of preoperative pulmonary testing in risk stratification and perioperative management. This study aims to evaluate the association between preoperative pulmonary function parameters and postoperative outcomes in patients undergoing open TAAA repair.

## 2. Materials and Methods

### 2.1. Study Design and Patient Selection

This study was conducted as a retrospective cohort analysis of patients who underwent open TAAA repair at our institution between 2006 and 2024. The study was approved by the Ethics Committee of the University Hospital RWTH Aachen (EK004/14) and was designed according to the Strengthening the Reporting of Observational Studies in Epidemiology (STROBE) guidelines and the Declaration of Helsinki [8]. Data entry into our registry occurred in two phases. For patients treated prior to formal protocol approval in 2020, clinical data were collected via retrospective chart review by experienced researchers. Following protocol approval in 2020, data for all subsequent patients were entered into the registry prospectively at the time of clinical care. Following approval, written informed consent was obtained from all patients. For patients who were unable to provide consent due to the severity of their illness and who had no available family members to provide surrogate consent, a waiver of consent was granted by the institutional review boards. A waiver of consent to collect retrospective data was also approved by the institutional review boards.

### 2.2. Inclusion Criteria

All patients undergoing elective or emergency open surgical repair for TAAA were eligible for inclusion if they had at least one parameter of pulmonary function testing available. Also patients with genetically triggered TAAA, including Marfan syndrome and other inherited aortopathies, were included even if they had different lung involvement [9].

### 2.3. Exclusion Criteria

Patients were excluded if preoperative pulmonary function testing data were unavailable or not measured. Mycotic (infected) thoracoabdominal aortic aneurysms were excluded from analysis. In cases where the distinction between covered/contained rupture and symptomatic TAAA could not be definitively established, clinical judgment and multidisciplinary consensus informed the final classification.

The definitive treatment plan for each patient was determined collaboratively by a multidisciplinary team comprising vascular surgeons, cardiac surgeons, interventional radiologists, cardiologists, and anesthesiologists. Endovascular repair options were not considered appropriate at the time of clinical decision-making due to anatomical factors, complexity of the aneurysm extent, or lack of suitable morphology for endovascular repair.

### 2.4. Surgery

The surgical protocol for open thoracoabdominal aortic aneurysm (TAAA) repair in our centre has been documented before [10]. All procedures were performed entirely by a dedicated team of specialized vascular surgeons with extensive training in complex open aortic reconstructions. The procedure includes double-lumen tube intubation with cerebrospinal fluid drainage (CSFD), perioperative monitoring of motor evoked potentials (MEPs), and positioning the patient on a beanbag in a modified right lateral decubitus position. The operating table was elongated to facilitate optimal access to the thoracic cavity. The surgical approach involved sequential aortic clamping when feasible, femorofemoral extracorporeal circulation (ECC) with distal aortic perfusion, selective visceral perfusion, and mild hypothermia (32–33 °C). Following complete heparinization (3 mg/kg; activated clotting time [ACT] maintained above 450 s), extracorporeal circulation (ECC) and distal aortic perfusion were initiated. Custodiol has been employed for renal perfusion instead of blood perfusion since 2014. Depending on the aneurysm’s extent, surgical access via thoracolaparotomy through the sixth to eighth intercostal space was adopted, and aortic reconstruction proceeded from proximal to distal and in cases of dissection from distal to proximal [1,10].

### 2.5. Definitions

Lung function operability thresholds were established following German clinical guidelines for thoracic surgery in lung cancer patients [11]. Patients were considered to have adequate pulmonary reserve if forced expiratory volume in one second (FEV1) exceeded 1.5 L, which represents the benchmark for lobectomy procedures. The more stringent threshold of FEV1 above 2.0 L, corresponding to pneumectomy criteria, was used as a secondary reference point. Because there are currently no validated absolute lung function thresholds specific to open TAAA repair, absolute thresholds (1.5 L and 2.0 L) were deliberately borrowed from German clinical guidelines for thoracic oncology. Ventilation patterns were classified according to current German recommendations for pulmonary function diagnostics [12]. An obstructive ventilation pattern was defined as FEV1 and forced vital capacity (FVC) both falling below the lower limit of normal (LLN). A restrictive ventilation pattern was characterized by total lung capacity (TLC) and vital capacity (VC) measurements below the LLN. Resting lung hyperinflation was identified when the Global Lung Initiative-derived residual volume z-score reached or exceeded 1.645. Emergency or urgent intervention was defined as surgical treatment initiated within 24 h of presentation due to symptomatic TAAA (characterized by severe thoracic, abdominal, or back pain) in the absence of frank aortic rupture, particularly in cases with an aortic diameter exceeding 50 mm and no alternative clinical explanation for presenting symptoms. Acute kidney injury (AKI) within 48 h postoperatively was defined according to the Kidney Disease Improving Global Outcomes (KDIGO) criteria based on serum creatinine levels [13]. In-hospital mortality was defined as any death within 30 days of surgery or during hospital stay. Pulmonary outcomes included pneumonia, duration of mechanical ventilation, and acute respiratory distress syndrome [14,15]. Cardiac complications included myocardial infarction and atrial fibrillation.

### 2.6. Statistics

Data are summarized using standard descriptive statistics, including frequencies, percentages, means, medians, standard deviations and quartiles, and visualized using scatter plots and boxplots. To assess association with duration of ventilation, Spearman’s correlation coefficients were calculated. Differences in proportions of outcome were explored using Chi-squared test and Fisher’s exact test. Nominal *p* values unadjusted for multiple testing are presented. Additionally, multivariable linear regression models were used to estimate adjusted differences in lung function parameters between patients with and without complications, and partial Spearman correlation coefficients were calculated to evaluate associations between lung function and ventilation time after adjustment for age, smoking status, COPD, emergency operation and time period. All analyses were performed on complete cases; no imputation was applied. Overall, 87% of patients had complete PFT data. Missing values were predominantly observed for body plethysmographic parameters (FRC %LLN and RV %LLN, ~12%), while spirometric parameters were nearly complete (FEV_1_ missing in <1%). Statistical analyses were carried out using R version 4.4.1. [R Core Team (2024) (Vienna, Austria). R: A language and environment for statistical computing. R Foundation for Statistical Computing, Vienna, Austria. URL https://www.R-project.org/.]

## 3. Results

Between 2006 and 2024, a total of 205 patients underwent open thoracoabdominal aortic aneurysm repair at our institution and had at least one preoperative pulmonary function testing parameter available for analysis. The cohort comprised predominantly male patients (72%) with a mean age of 55.3 years at the time of surgery. The baseline demographic and clinical characteristics are summarized in Table 1.

**Table 1:** Baseline Demographics and Clinical Characteristics (N = 205).

Characteristic	Value
**Demographics**	
Age (years), mean ± SD	55.3 ± 11.8
Height (cm), mean ± SD	178.3 ± 10.4
Weight (kg), mean ± SD	83.9 ± 17.8
Body mass index (kg/m^2^), mean ± SD	26.3 ± 4.5
Male sex, n (%)	148 (72.2%)
**Smoking History, n (%)**	
Never smoker	118 (57.6%)
Current smoker	65 (31.7%)
Former smoker	22 (10.7%)
**Genetically triggered aortopathies, n (%)**	40 (19.5%)
Marfan syndrome	37 (92.5%)
Ehlers–Danlos syndrome	1 (2.5%)
Loeys–Dietz syndrome	1 (2.5%)
Moyamoya syndrome	1 (2.5%)
**Cardiovascular Comorbidities, n (%)**	
Arterial hypertension	192 (93.7%)
Coronary artery disease	46 (22.4%)
Congestive heart failure	94 (45.9%)
- NYHA Class I	72 (76.6%)
- NYHA Class II	13 (13.8%)
- NYHA Class III	9 (9.6%)
Atrial fibrillation	17 (8.3%)
Ischemic cardiomyopathy	5 (2.4%)
Previous myocardial infarction	4 (2.0%)
Peripheral arterial disease	11 (5.4%)
Pulmonary hypertension	1 (0.5%)
**Other Comorbidities, n (%)**	
Pre-existing lung disease	87 (42.4%)
- Obstructive	54 (26.3%)
- Restrictive	18 (8.8%)
- Mixed obstructive/restrictive	15 (7.3%)
Obesity (BMI ≥30 kg/m^2^)	37 (18.0%)
Hypercholesterolemia	105 (51.2%)
Diabetes mellitus	13 (6.3%)
Chronic kidney disease	29 (14.1%)
Previous stroke	23 (11.2%)
**Previous Cardiovascular Interventions, n (%)**	
Previous aortic surgery (total)	119 (58.0%)
- Prior ascending aorta/aortic arch repair	57 (27.8%)
- Prior descending thoracic aorta repair	37 (18.0%
- Prior thoracoabdominal aorta repair	21 (10.2%)
- Prior abdominal aorta repair	20 (9.8%)
Previous TEVAR	33 (16.1%)
Percutaneous coronary intervention	54 (26.3%)
Coronary artery bypass grafting	9 (4.4%)
Aortic valve surgery	50 (24.4%)
- Mechanical valve	17 (8.3%)
- Biological valve	11 (5.4%)
- Valve reconstruction	9 (4.4%)
Pacemaker implantation	6 (2.9%)
Bypass surgery (peripheral/visceral)	30 (14.6%)
**Preoperative Medications, n (%)**	
Antihypertensive therapy	111 (54.1%)
Statin therapy	42 (20.5%)
Aspirin monotherapy	54 (26.3%)
Dual antiplatelet therapy	3 (1.5%)
Anticoagulation	33 (16.1%)

Data are presented as mean ± standard deviation or n (%). NYHA = New York Heart Association; TEVAR = thoracic endovascular aortic repair; BMI = body mass index. An overview of preoperative pulmonary function test results can be found in Table 2.

**Table 2:** Preoperative Pulmonary Function Test Results.

Parameter	n	Mean ± SD	Median	Range (Min–Max)
**Spirometry**				
Vital capacity (VC), L	192	3.79 ± 0.98	3.89	0.78–5.80
Vital capacity (VC), % LLN	186	96.9 ± 20.5	97.5	26.3–233.9
FEV_1_, L	202	2.72 ± 0.79	2.66	0.72–5.11
FEV_1_, % LLN	195	99.6 ± 23.5	97.3	30.9–163.9
FEV_1_/FVC ratio, %	197	74.1 ± 9.1	75.2	47.0–98.4
**Body Plethysmography**				
Total lung capacity (TLC), L	186	6.72 ± 1.28	6.79	3.16–9.96
Total lung capacity (TLC), % LLN	181	120.5 ± 20.1	119.8	66.9–174.6
Functional residual capacity (FRC), L	185	3.86 ± 0.93	3.72	1.12–7.68
Functional residual capacity (FRC), % LLN	180	155.4 ± 33.6	154.2	54.7–268.1
Residual volume (RV), L	185	2.98 ± 0.86	2.91	0.65–7.11
Residual volume (RV), % LLN	180	245.8 ± 65.0	245.7	45.8–457.2

FEV_1_ = forced expiratory volume in 1 second; FVC = forced vital capacity; LLN = lower limit of normal. Data are presented as mean ± standard deviation, n (%), or as indicated.

### 3.1. Spirometry and Body Plethysmography and Postoperative Outcomes

Postoperative pulmonary complications occurred in 126 patients (61.5%), with pneumonia in 96 patients (46.8%) and acute respiratory distress syndrome (ARDS) in 31 patients (15.1%). Spearman correlation analysis revealed significant inverse associations between preoperative spirometric parameters and duration of mechanical ventilation. Lower preoperative vital capacity (ρ = −0.218, *p* = 0.017), vital capacity % LLN (ρ = −0.288, *p* = 0.002), FEV_1_ (ρ = −0.205, *p* = 0.021), and FEV_1_ % LLN (ρ = −0.286, *p* = 0.001) were each associated with prolonged postoperative mechanical ventilation. No significant correlations were observed between ventilation time and functional residual capacity, residual volume, or total lung capacity (all *p* > 0.05). When comparing patients who developed any pulmonary complication to those who did not, no statistically significant differences were observed in absolute lung function parameters.

**Figure 1:**
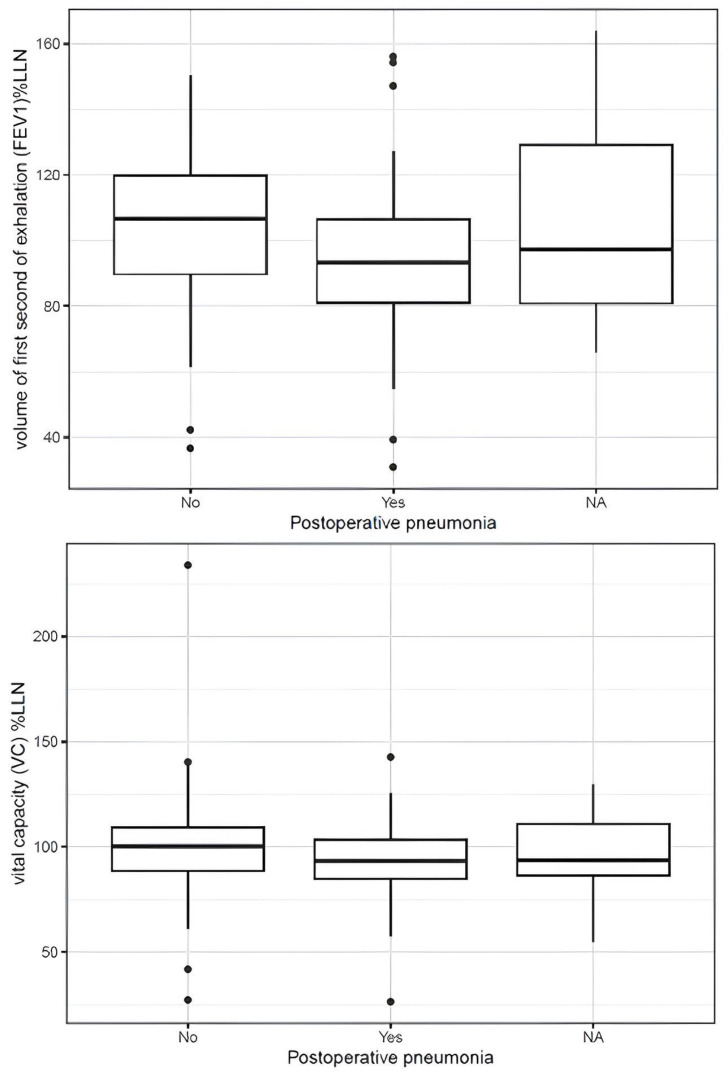
Preoperative vital capacity (VC %LLN) and forced expiratory volume in 1 second (FEV_1_ %LLN), both expressed as percentage of the lower limit of normal, stratified by postoperative pneumonia. The LLN was adjusted for age, sex, and height. The horizontal line represents the median; boxes represent the interquartile range; whiskers extend to 1.5× the interquartile range; dots represent outliers.

Patients who developed postoperative pneumonia exhibited significantly lower preoperative vital capacity when expressed as percentage of predicted (93.90 ± 16.36%LLN vs. 100.48 ± 24.68%LLN; mean difference −6.58, 95% CI −13.01 to −0.15, *p* = 0.045). FEV_1_ %LLN was also significantly lower in pneumonia patients (94.53 ± 21.69 vs. 104.53 ± 23.15; mean difference −10.01, 95% CI −16.81 to −3.20, *p* = 0.004) (Figure 1). Absolute spirometric values and body plethysmographic parameters showed no significant associations with pneumonia risk.

**Figure 2:**
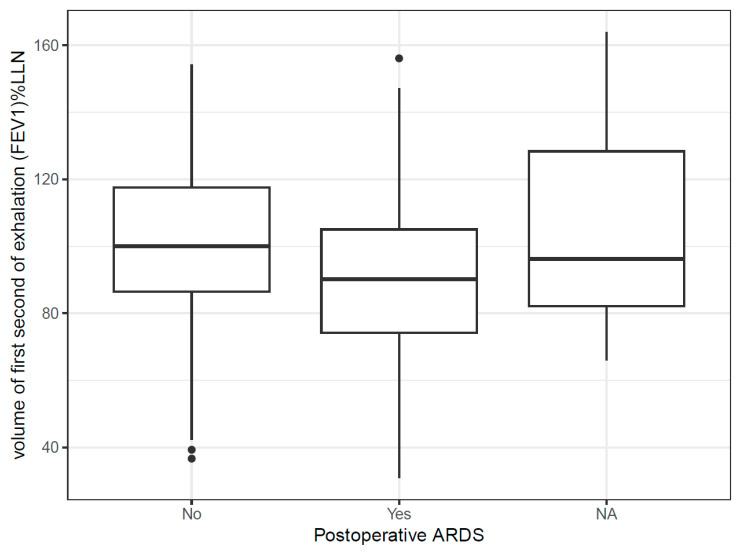
Preoperative FEV_1_ expressed as percentage of the lower limit of normal (FEV_1_ %LLN) stratified by postoperative ARDS. The LLN was adjusted for age, sex, and height. The horizontal line represents the median; boxes represent the interquartile range; whiskers extend to 1.5× the interquartile range; dots represent outliers.

Among patients who developed postoperative ARDS, preoperative FEV_1_ %LLN was lower (92.13 ± 25.80 vs. 100.65 ± 22.01; mean difference −8.53, 95% CI −17.45 to 0.40, *p* = 0.061). A similar trend was observed for VC %LLN (90.13 ± 19.51 vs. 98.46 ± 21.01; mean difference −8.32, 95% CI −16.85 to 0.20, *p* = 0.056) (Figure 2). Absolute lung function values and body plethysmographic measurements demonstrated no significant differences between groups.

### 3.2. Other Complications

Cardiac complications occurred in 57 patients (27.8%). Patients who developed cardiac complications exhibited significantly higher preoperative residual volume (3.16 ± 0.85 L vs. 2.87 ± 0.79 L, *p* = 0.021). No significant differences were observed in spirometric parameters, including vital capacity, FEV_1_, or FEV_1_/FVC ratio (all *p* > 0.05). Total lung capacity and other body plethysmographic measurements showed no significant associations with cardiac morbidity. No significant associations were identified between preoperative lung function parameters and the development of neurological complications (all *p* > 0.05).

**Figure 3:**
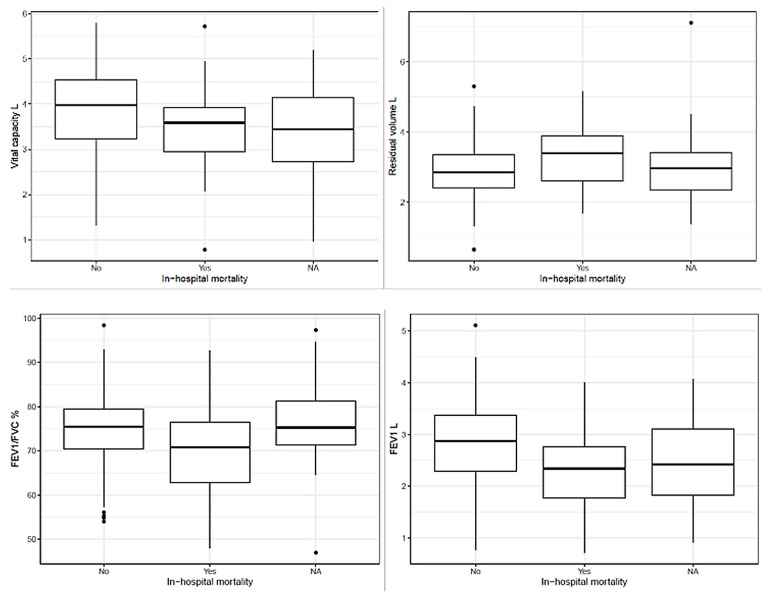
Preoperative lung function parameters stratified by in-hospital mortality. Box plots display the distribution of vital capacity (VC, litres), forced expiratory volume in 1 second (FEV_1_, litres), FEV_1_/FVC ratio (%), and residual volume (RV, litres) in survivors (No) and non-survivors (Yes). The horizontal line represents the median; boxes represent the interquartile range; whiskers extend to 1.5× the interquartile range; dots represent outliers.

In-hospital mortality occurred in 25 patients (12.2%). Notably, no patients in this cohort were discharged and subsequently died within 30 days of surgery. Non-survivors demonstrated significantly lower preoperative vital capacity (3.46 ± 1.05 L vs. 3.92 ± 0.92 L; mean difference −0.47, 95% CI −0.88 to −0.05, *p* = 0.028) and markedly reduced FEV_1_ (2.30 ± 0.74 L vs. 2.84 ± 0.76 L; mean difference −0.54, 95% CI −0.86 to −0.22, *p* = 0.001). The FEV_1_/FVC ratio was significantly lower in patients who died (68.80 ± 11.37% vs. 74.61 ± 8.09%; mean difference −5.81, 95% CI −9.57 to −2.05, *p* = 0.003). Non-survivors also exhibited higher residual volume (3.37 ± 0.90 L vs. 2.90 ± 0.79 L; mean difference 0.48, 95% CI 0.12 to 0.83, *p* = 0.010) (Figure 3).

### 3.3. Ventilation Patterns and Postoperative Outcomes

Patients were classified into obstructive ventilation patterns (n = 85, 45.2%), restrictive ventilation patterns (n = 26, 14.1%), and resting lung hyperinflation (n = 56, 30.3%). Obstructive patterns were significantly associated with acute kidney injury (*p* = 0.044) but not with pulmonary, cardiac, or neurological complications. Restrictive patterns predicted ARDS (*p* = 0.040) and acute kidney injury (*p* = 0.043). Hyperinflation was significantly associated with atrial fibrillation (*p* = 0.039) and stroke (*p* = 0.034). Traditional operability thresholds demonstrated that FEV_1_ below 2.0 L predicted mortality (*p* = 0.037), while FEV_1_ below 1.5 L predicted acute kidney injury (*p* = 0.017), spinal cord ischemia (*p* = 0.035), and mortality (*p* = 0.023). The majority of patients (96.0%) met the 1.5 L criteria for operability (Table 3).

### 3.4. Pulmonary Function Testing and Long-Term Mortality

Follow-up data were available for 142 of 205 patients (69.3%) with a median survival of 6.35 years; 54 deaths occurred during follow-up. Cox proportional hazards regression identified FEV_1_ and the FEV_1_/FVC ratio as significant predictors of long-term mortality, with patients in the highest FEV_1_ quartile (3.31–5.11 L) demonstrating substantially reduced mortality risk (HR 0.27, 95% CI 0.10–0.73, *p* = 0.01) and those with the highest FEV_1_/FVC ratio showing protective effects (HR 0.34, 95% CI 0.14–0.86, *p* = 0.02). There was also a trend for elevated residual volume predicting worse survival outcomes (HR 1.91 for highest quartile, *p* = 0.08), although it did not reach statistical significance. Vital capacity, functional residual capacity, total lung capacity, and categorical ventilation pattern classifications showed no significant associations with long-term mortality.

### 3.5. Subgroup Analysis

Subgroup analyses stratified by genetically triggered aortopathy (GTA; 40/205, 19.5%) showed that GTA status did not show differences between preoperative pulmonary function and postoperative outcomes. In non-GTA patients, lower VC, VC LLN, FEV_1_ and FEV_1_ LLN were significantly associated with longer duration of mechanical ventilation (e.g., VC LLN ρ = −0.27, *p* = 0.007; FEV_1_ LLN ρ = −0.23, *p* = 0.021), whereas in GTA patients, only FEV_1_ LLN remained significantly correlated with ventilation time (ρ = −0.44, *p* = 0.024). Reduced FEV_1_ LLN was significantly associated with postoperative pneumonia in non-GTA patients (*p* = 0.009; VC LLN borderline, *p* = 0.054), but not in GTA patients. For ARDS, lower FRC was associated with ARDS in non-GTA patients (*p* = 0.029), while in GTA patients, lower VC distinguished those who developed ARDS (VC *p* = 0.035).

Cardiac complications in non-GTA patients were linked to higher RV (*p* = 0.019), with a similar hyperinflation pattern in GTA patients where FRC LLN remained significantly higher in those with cardiac events (*p* = 0.03). Atrial fibrillation in both groups was associated with hyperinflation (elevated RV/RVLLN and higher TLC/TLC LLN, all *p* < 0.05), whereas continuous lung function parameters showed no consistent association with acute kidney injury, spinal cord ischemia or stroke in either group beyond the pattern already seen in the overall cohort. For in-hospital mortality, non-survivors in both GTA and non-GTA strata exhibited lower VC and FEV_1_ and higher RV; these differences reached statistical significance predominantly in the larger non-GTA subgroup (e.g., FEV_1_ *p* = 0.002, VC *p* = 0.039, RV *p* = 0.019).

### 3.6. Multivariable Analyses

Multivariable analyses were performed adjusting for age, smoking status, COPD, emergency operation, and time period (Supplementary Material Tables S1 and S2). After adjustment, FEV_1_ %LLN remained significantly lower in patients who developed pneumonia (adjusted difference −5.84, *p* = 0.038) and ARDS (−8.33, *p* = 0.021) and in those who died in hospital (−11.07, *p* = 0.007). VC %LLN was independently associated with in-hospital mortality (−9.13, *p* = 0.044) and showed a trend for ARDS (−7.04, *p* = 0.081). Partial Spearman correlations confirmed that VC %LLN remained significantly correlated with prolonged mechanical ventilation after adjustment (ρ = −0.224, *p* = 0.014), while the association for FEV_1_ %LLN showed a trend (ρ = −0.175, *p* = 0.071). Associations between lung function parameters and cardiac or neurologic complications remained non-significant, consistent with the unadjusted analyses.

## 4. Discussion

This study evaluated the association between preoperative pulmonary function and postoperative complications in 205 patients undergoing open TAAA repair. Using a combination of spirometry and body plethysmography, we identified that reduced preoperative lung function, particularly when expressed as percentage of predicted values, is associated with major postoperative morbidity and mortality. Notably, preoperative vital capacity and FEV1, when normalized to age-, height-, and sex-adjusted lower limits of normal, were significantly associated with pneumonia, ARDS, and in-hospital mortality. These findings challenge traditional operability thresholds and support a shift toward precision-medicine approaches.

Reduced FEV_1_ %LLN was significantly associated with postoperative pneumonia and ARDS, and these associations persisted after multivariable adjustment (adjusted *p* = 0.039 and *p* = 0.020, respectively). This aligns with thoracic surgery literature demonstrating that percentage predicted values provide superior risk stratification compared to absolute measurements [16,17,18]. Recently, Girardi et al. reported that preoperative FEV_1_ < 50% strongly predicted respiratory failure, tracheostomy requirement, and operative mortality in 726 patients undergoing open thoracic aortic repair [19]. Our findings extend this literature by demonstrating that percentage predicted values discriminate risk even when the majority of patients exceed traditional operability thresholds. Also, long-term survival analysis showed that absolute FEV_1_ and the FEV_1_/FVC ratio independently predicted mortality during follow-up. Elevated residual volume was associated with worse survival, reflecting impaired cardiac function from hyperinflation. Ventilation patterns, however, did not predict long-term mortality despite their demonstrated association with acute perioperative complications, suggesting they primarily influence short-term morbidity rather than long-term outcomes [20,21].

The mechanisms underlying the association between reduced lung function and respiratory complications in TAAA repair are multifactorial. The surgical approach necessitates prolonged single-lung ventilation, creating substantial ventilation–perfusion mismatch and promoting atelectasis [22]. Patients with an already compromised baseline reserve—reflected by lower FEV_1_ and vital capacity—possess limited ability to compensate for this acute event [19,23]. Second, impaired expiratory flow rates and reduced lung volumes compromise effective cough and airway clearance in the postoperative period, predisposing to mucus plugging, pneumonia, and progressive respiratory failure [24]. Third, the extensive surgical operation and obligatory cross-clamping of the descending aorta trigger systemic inflammatory responses that may culminate in acute lung injury, particularly in patients with preexisting pulmonary pathology [22,25]. The elevated rate of pneumonia in our cohort is probably driven by our broad diagnostic criteria, as any new positive pulmonary infiltrate on routine postoperative imaging was classified as pneumonia. Consequently, some cases of severe atelectasis related to prolonged single-lung ventilation may have been captured within this endpoint.

The correlation between reduced preoperative lung function and prolonged mechanical ventilation persisted for VC %LLN after multivariable adjustment (partial ρ = −0.224, *p* = 0.014), while a trend with FEV_1_ %LLN remained (partial ρ = −0.175, *p* = 0.071). Although these correlations are modest, they could be clinically relevant. In practice, this association could help identify vulnerable patients who may benefit from targeted perioperative strategies. Extended ventilator dependence not only increases pneumonia risk through ventilator-associated mechanisms but also compounds the likelihood of developing ARDS, creating a vicious cycle of pulmonary deterioration [26].

Beyond respiratory outcomes, we also found unexpected associations with several other postoperative complications not typically considered pulmonary in etiology. Both obstructive and restrictive ventilation patterns showed a significant difference in AKI stage distribution (*p* = 0.044 and *p* = 0.043). These findings however must be interpreted with caution; zero events in certain subgroups precluded OR estimation, these are nominal unadjusted *p*-values, and the associations were not confirmed after multivariable adjustment. While the literature describes ‘lung–kidney crosstalk’—whereby compromised pulmonary reserve, prolonged mechanical ventilation, and systemic inflammation can exacerbate renal hypoperfusion [27,28]—the main cause of AKI in open TAAA repair is most probably profound intraoperative insults, including aortic cross-clamping, direct renal ischemia–reperfusion injury, surgical blood loss, and hemodynamic fluctuations, compounded by a systemic inflammatory response promoting renal vasoconstriction and microvascular dysfunction [29,30,31,32,33]. Although 96% of patients met traditional operability thresholds (FEV_1_ > 1.5 L), absolute FEV_1_ below 2.0 L and 1.5 L remained significant predictors of mortality (*p* = 0.037 and *p* = 0.023) and multi-organ complications. As these cut-offs are historically borrowed from thoracic oncology and lack validation in TAAA repair, their routine clinical application for aortic surgery has been questioned. Our results confirm that relying on these unvalidated absolute thresholds is inadequate for TAAA candidates [21].

Additionally, the relatively young mean age of our cohort reflects modern patient selection, where older degenerative patients are managed endovascularly and open repair is concentrated among younger patients with genetically triggered aortopathies (19.5% of this cohort) or complex post-dissection anatomies [34]. Our subgroup analysis is consistent with the known pulmonary differences of genetically triggered aortopathies, in which intrinsic lung involvement and altered chest wall mechanics (e.g., pectus deformities, scoliosis) predispose to restrictive ventilatory patterns and high pulmonary complication rates [35,36]. In this context, it is plausible that reduced VC was particularly predictive of ARDS in GTA patients, whereas FRC and other parameters predominated in non-GTA individuals. Importantly, the overall direction and magnitude of lung function–outcome associations were similar in both groups, suggesting that GTA status does not fundamentally modify the prognostic value of preoperative pulmonary function testing. While the long study period includes a major shift in aortic surgery characterized by the rise of endovascular techniques, a major strength of this study is the consistency of our institutional approach. As we have previously demonstrated, while the widespread adoption of endovascular repair shifted the open surgical case-mix toward younger patients with genetic aortopathies and complex post-dissection anatomies, our standardized perioperative and surgical protocols remained unchanged. This minimizes the risk of confounding related to evolving perioperative care.

These results could have implications for preoperative assessment and preoperative management of TAAA candidates. Rather than applying uniformly to all patients, as discussed above, the demonstrated associations between ventilation patterns and specific complications might enable precision medicine. Patients with restrictive patterns and higher ARDS risk should be prioritized for pulmonary rehabilitation, as preoperative programs have been shown to reduce postoperative pulmonary complications and improve exercise capacity and respiratory muscle strength [37,38,39]. Early postoperative extubation should be pursued wherever feasible. In patients with obstructive or hyperinflation patterns, targeted strategies—including stringent smoking cessation (ideally ≥2 weeks before surgery), preoperative inspiratory muscle training, and pharmacological optimization with bronchodilators, inhaled corticosteroids, and systemic steroids in COPD or asthma—are essential to maximize lung function and may mitigate the excess risks of renal and cardiac complications identified in this cohort [40,41,42].

Comparing our findings to cardiac surgery literature provides important parallels. Kuwata and colleagues demonstrated that preoperative testing predicted all complications including respiratory complications after cardiac surgery [43]. McAllister et al.’s retrospective analysis of cardiac surgery patients found that each 10% decrease in preoperative FEV_1_ % predicted increased mortality risk by 10%, validating our findings [21].

Some important limitations of this study need to be acknowledged. The retrospective design precludes causal inference and selection bias plays a role; excluding patients deemed non-operable may attenuate associations. Also, the small event numbers for several of the outcomes reduce statistical power. Not all patients had complete spirometric and plethysmographic data, potentially introducing measurement bias. Unmeasured confounding from intraoperative variables (single-lung ventilation duration, cross-clamp time, transfusion volume) and patient frailty cannot be excluded. Additionally, our classification of ventilation patterns was based on national German guidelines reflecting our institutional clinical routine, which marginally differs from the most recent ATS/ERS technical standards for interpreting routine lung function tests [44]. And the association between ventilation patterns and some of the outcomes must be interpreted with caution, as odds ratio estimation was not possible due to sparse events, and dominant intraoperative factors were not adjusted for. Finally, our study spans 24 years during which complex endovascular TAAA repair emerged and became increasingly adopted. This likely altered referral patterns and patient selection for open repair; however, outcomes remained the same over the years [34]. Long-term follow-up was available for 69.3% of patients. Despite efforts to contact all surviving patients, loss to follow-up, partly due to the high baseline mortality of TAAA and the wide geographic referral pattern of specialized aortic centers, could have introduced bias.

Nevertheless, our findings argue against traditional thresholds derived from lung resection surgery and support an individual-based risk stratification framework that integrates percentage-predicted spirometry, ventilation patterns, and hyperinflation. In clinical practice, such a framework could help identify patients who warrant intensified perioperative monitoring and targeted preoperative prehabilitation.

## 5. Conclusions

Preoperative pulmonary function assessment may help identify TAAA patients at increased risk of postoperative complications and mortality. Combining percentage-predicted spirometry, ventilation patterns, and hyperinflation markers may support individualized treatment selection, prehabilitation, and perioperative monitoring based on each patient’s specific risk profile.

## Figures and Tables

**Table 3:** Association Between Preoperative Ventilation Patterns and Postoperative Outcomes.

Outcome	Obstructive Pattern (n = 85)	Restrictive Pattern (n = 26)	Resting Hyperinflation (n = 56)	FEV_1_ >2.0 L (n = 163)	FEV_1_ >1.5 L (n = 194)
Pulmonary Complications					
Any pulmonary complication	1.33 (0.67–2.66), *p* = 0.519	1.70 (0.63–5.39), *p* = 0.451	1.27 (0.60–2.82), *p* = 0.682	0.66 (0.23–1.65), *p* = 0.198	0.63 (0.03–4.42), *p* = 0.065
Pneumonia	1.69 (0.91–3.18), *p* = 0.133	1.53 (0.63–3.86), *p* = 0.477	1.17 (0.58–2.35), *p* = 0.797	0.63 (0.26–1.44), *p* = 0.277	0.81 (0.10–5.00), *p* = 0.111
ARDS	2.03 (0.91–4.69), *p* = 0.128	2.88 (1.05–7.49), ***p* = 0.040**	2.05 (0.84–4.88), *p* = 0.162	1.41 (0.49–5.09), *p* = 0.659	0.89 (0.13–17.72), *p* = 0.278
Cardiac Complications					
Any cardiac complication	0.80 (0.41–1.53), *p* = 0.607	0.81 (0.29–2.01), *p* = 0.832	1.44 (0.70–2.94), *p* = 0.420	0.88 (0.38–2.12), *p* = 0.668	0.32 (0.04–1.99), *p* = 0.295
Myocardial infarction	1.04 (0.29–3.60), *p* = 0.657	†, *p* = 0.561	†, *p* = 0.632	0.49 (0.13–2.37), *p* = 0.315	†, *p* = 1.000
Atrial fibrillation	0.25 (0.04–1.18), *p* = 0.122	0.13 (0.01–1.60), *p* = 0.148	†, ***p* = 0.039**	0.72 (0.03–5.83), *p* = 0.777	†, *p* = 0.235
Renal Complications					
Acute kidney injury	†, ***p* = 0.044**	†, ***p* = 0.043**	†, *p* = 0.354	†, *p* = 0.100	†, ***p* = 0.017**
Neurological Complications					
Any neurological complication	1.00 (0.53–1.87), *p* = 1.000	1.19 (0.48–2.86), *p* = 0.873	1.59 (0.79–3.21), *p* = 0.261	0.79 (0.35–1.82), *p* = 0.455	0.92 (0.15–7.11), *p* = 0.182
Spinal cord ischemia	1.79 (0.59–5.71), *p* = 0.531	2.84 (0.71–9.76), *p* = 0.251	1.06 (0.28–3.49), *p* = 1.000	1.10 (0.27–7.38), *p* = 0.056	0.19 (0.02–4.15), ***p* = 0.035**
Stroke	1.04 (0.29–3.60), *p* = 1.000	3.69 (0.90–13.40), *p* = 0.063	†, ***p* = 0.034**	†, *p* = 1.000	†, *p* = 0.680
Mortality					
In-hospital mortality	1.17 (0.48–2.84), *p* = 0.906	1.23 (0.33–3.70), *p* = 0.753	1.05 (0.38–2.68), *p* = 1.000	0.34 (0.13–0.92), ***p* = 0.037**	0.10 (0.01–0.65), ***p* = 0.023**

Values represent *p*-values from chi-square or Fisher’s exact test. † OR not estimable due to zero events in one of the subgroups. Bold values indicate statistical significance (*p* < 0.05).

## Data Availability

Data availability on request.

## Supplementary Materials

The following supporting information can be downloaded at: https://www.mdpi.com/article/10.3390/jcm15072623/s1, Table S1: Adjusted difference in lung function parameters between presence/absence of complications (adjusted for age, smoking status, COPD, emergency operation and time period); Table S2: Partial Spearman correlation coefficients between lung function parameters and ventilation time (adjusted for age, smoking status, COPD, emergency operation and time period).
