# Supplementary material for: Pulmonary Risk Stratification in Open Thoracoabdominal Aortic Aneurysm Repair
Source: J Clin Med. 2026 Mar 30;15(7):2623. doi: 10.3390/jcm15072623 (PMC13072977; doi:10.3390/jcm15072623)
Supplement: Supplementary file 1 [file jcm-15-02623-s001.zip › jcm-4153789-Supplementary Table S1.pdf]

Supplementary Table S1: Adjusted difference in lung function parameters between presence/absence of complications (adjusted for age, smoking status, COPD, emergency operation and time period)

Pulmonary complications

	Estimate	Std. Error	t value	Pr(>|t|)	2.5 %	97.5 %
vital capacity (VC)	-0.012	0.167	-0.071	0.943	-0.342	0.318
vital capacity (VC) %LLN	-3.181	3.480	-0.914	0.362	-10.057	3.695
functional residual capacity (FRC)	0.136	0.163	0.834	0.405	-0.187	0.459
functional residual capacity (FRC)%LLN	1.783	5.597	0.319	0.751	-9.277	12.842
residual volume (RV)	0.066	0.137	0.482	0.630	-0.205	0.337
residual volume (RV)%LLN	-2.078	11.368	-0.183	0.855	-24.543	20.388
total lung capacity (TLC)	0.069	0.233	0.297	0.767	-0.392	0.530
total lung capacity (TLC) %LLN	-3.069	3.285	-0.934	0.352	-9.561	3.423
FEV 1 % VC IN (FEV1/FVC)	0.371	1.388	0.267	0.790	-2.370	3.111
FEV1	-0.055	0.124	-0.443	0.659	-0.299	0.190
FEV1 %LLN	-4.343	3.227	-1.346	0.180	-10.715	2.029

Pneumonia

	Estimate	Std. Error	t value	Pr(>|t|)	2.5 %	97.5 %
vital capacity (VC)	-0.070	0.147	-0.477	0.634	-0.360	0.220
vital capacity (VC) %LLN	-4.981	3.051	-1.633	0.105	-11.008	1.046
functional residual capacity (FRC)	-0.015	0.145	-0.101	0.920	-0.301	0.272
functional residual capacity (FRC)%LLN	-4.214	4.937	-0.854	0.395	-13.970	5.541
residual volume (RV)	0.058	0.121	0.482	0.631	-0.181	0.298
residual volume (RV)%LLN	-0.778	10.047	-0.077	0.938	-20.632	19.076
total lung capacity (TLC)	-0.012	0.206	-0.058	0.953	-0.420	0.395
total lung capacity (TLC) %LLN	-3.554	2.896	-1.227	0.222	-9.277	2.169
FEV 1 % VC IN (FEV1/FVC)	-0.955	1.219	-0.784	0.435	-3.363	1.453
FEV1	-0.093	0.108	-0.865	0.388	-0.306	0.119
FEV1 %LLN	-5.839	2.793	-2.090	0.038	-11.355	-0.322

ARDS

	Estimate	Std. Error	t value	Pr(>|t|)	2.5 %	97.5 %
vital capacity (VC)	-0.155	0.194	-0.799	0.426	-0.537	0.228
vital capacity (VC) %LLN	-7.043	4.010	-1.756	0.081	-14.966	0.879
functional residual capacity (FRC)	0.282	0.190	1.480	0.141	-0.094	0.657
functional residual capacity (FRC)%LLN	6.511	6.550	0.994	0.322	-6.434	19.455
residual volume (RV)	0.237	0.160	1.484	0.140	-0.078	0.552
residual volume (RV)%LLN	9.894	13.331	0.742	0.459	-16.452	36.240
total lung capacity (TLC)	-0.020	0.271	-0.074	0.941	-0.556	0.516
total lung capacity (TLC) %LLN	-4.251	3.819	-1.113	0.267	-11.797	3.295

FEV 1 % VC IN (FEV1/FVC)	0.954	1.598	0.597	0.552	-2.203	4.111
FEV1	-0.173	0.138	-1.251	0.213	-0.446	0.100
FEV1 %LLN	-8.329	3.568	-2.334	0.021	-15.376	-1.283

Cardiac complications

	Estimate	Std. Error	t value	Pr(>|t|)	2.5 %	97.5 %
vital capacity (VC)	-0.172	0.154	-1.120	0.264	-0.476	0.131
vital capacity (VC) %LLN	-3.412	3.219	-1.060	0.291	-9.770	2.946
functional residual capacity (FRC)	0.121	0.150	0.808	0.421	-0.176	0.418
functional residual capacity (FRC)%LLN	5.845	5.123	1.141	0.256	-4.279	15.968
residual volume (RV)	0.189	0.125	1.516	0.132	-0.057	0.435
residual volume (RV)%LLN	15.020	10.338	1.453	0.148	-5.409	35.449
total lung capacity (TLC)	0.020	0.214	0.094	0.925	-0.403	0.443
total lung capacity (TLC) %LLN	1.883	3.015	0.625	0.533	-4.075	7.842
FEV 1 % VC IN (FEV1/FVC)	-0.738	1.275	-0.579	0.564	-3.255	1.780
FEV1	-0.158	0.111	-1.417	0.158	-0.377	0.062
FEV1 %LLN	-4.751	2.911	-1.632	0.105	-10.499	0.997

Neurologic complications

	Estimate	Std. Error	t value	Pr(>|t|)	2.5 %	97.5 %
vital capacity (VC)	0.072	0.152	0.473	0.637	-0.228	0.371
vital capacity (VC) %LLN	2.787	3.175	0.878	0.381	-3.484	9.058
functional residual capacity (FRC)	0.011	0.150	0.074	0.941	-0.285	0.307
functional residual capacity (FRC)%LLN	1.438	5.112	0.281	0.779	-8.664	11.539
residual volume (RV)	-0.031	0.126	-0.246	0.806	-0.280	0.218
residual volume (RV)%LLN	-3.440	10.440	-0.330	0.742	-24.070	17.190
total lung capacity (TLC)	0.011	0.214	0.053	0.958	-0.411	0.434
total lung capacity (TLC) %LLN	0.075	3.018	0.025	0.980	-5.889	6.038
FEV 1 % VC IN (FEV1/FVC)	-0.967	1.247	-0.775	0.439	-3.430	1.497
FEV1	-0.015	0.110	-0.140	0.889	-0.233	0.202
FEV1 %LLN	-0.258	2.900	-0.089	0.929	-5.985	5.470

In hospital mortality

	Estimate	Std. Error	t value	Pr(>|t|)	2.5 %	97.5 %
vital capacity (VC)	-0.297	0.217	-1.371	0.172	-0.725	0.131
vital capacity (VC) %LLN	-9.131	4.504	-2.028	0.044	-18.028	-0.234
functional residual capacity (FRC)	0.025	0.215	0.115	0.909	-0.400	0.450
functional residual capacity (FRC)%LLN	-2.249	7.348	-0.306	0.760	-16.769	12.271
residual volume (RV)	0.187	0.176	1.061	0.291	-0.161	0.535
residual volume (RV)%LLN	17.455	14.578	1.197	0.233	-11.354	46.264
total lung capacity (TLC)	-0.048	0.302	-0.160	0.873	-0.645	0.548
total lung capacity (TLC) %LLN	-2.148	4.257	-0.505	0.615	-10.560	6.264

FEV 1 % VC IN (FEV1/FVC)	-2.515	1.800	-1.398	0.164	-6.070	1.039
FEV1	-0.264	0.157	-1.684	0.094	-0.574	0.046
FEV1 %LLN	-11.073	4.064	-2.725	0.007	-19.097	-3.049
