# Supplementary material for: Pulmonary Risk Stratification in Open Thoracoabdominal Aortic Aneurysm Repair
Source: J Clin Med. 2026 Mar 30;15(7):2623. doi: 10.3390/jcm15072623 (PMC13072977; doi:10.3390/jcm15072623)
Supplement: Supplementary file 1 [file jcm-15-02623-s001.zip › jcm-4153789-Supplementary Table S2.pdf]

Supplementary Table S2: Partial Spearman correlation coefficients between lung function parameters and ventilation time (adjusted for age, smoking status, COPD, emergency operation and time period)

	sp_rho	sp_var	p	ci_lower	ci_upper	n
VC	-0.121	0.009	0.217	-0.305	0.071	120
VC_LLN	-0.224	0.008	0.014	-0.387	-0.047	119
FRC	0.088	0.011	0.402	-0.118	0.288	114
FRC_LLN	0.027	0.011	0.794	-0.174	0.227	114
RV	0.035	0.010	0.729	-0.162	0.229	113
RV_LLN	-0.017	0.009	0.851	-0.197	0.163	113
TLC	-0.057	0.010	0.576	-0.251	0.142	114
TLC_LLN	-0.147	0.009	0.120	-0.323	0.039	114
FEVperFVC	0.067	0.012	0.535	-0.145	0.274	123
FEV1	-0.119	0.009	0.212	-0.298	0.068	128
FEV1_LLN	-0.175	0.009	0.071	-0.353	0.015	127
